# Long-Term Therapy with Long-Acting Lipoglycopeptide Antibiotics in the Treatment of Cardiovascular Prosthetic Infections: A Systematic Review

**DOI:** 10.3390/antibiotics14111130

**Published:** 2025-11-07

**Authors:** Francesca Gavaruzzi, Guido Granata, Alessandro Capone, Pierangelo Chinello, Stefania Cicalini

**Affiliations:** Clinical and Research Department for Infectious Diseases, Systemic and Immune Depression-Associated Infections Unit, National Institute for Infectious Diseases “Lazzaro Spallanzani”-IRCCS, 00149 Rome, Italy

**Keywords:** long-acting lipoglycopeptide antibiotics, cardiovascular prosthetic infections, dalbavancin, oritavancin, chronic suppressive antibiotic therapy, endocarditis outcome, mortality, cardiovascular infections, prosthetic valve endocarditis, cardiac implantable electronic device infections, vascular prostheses infections, left ventricular assist device infections, source control

## Abstract

**Background**: Dalbavancin and oritavancin are long-acting lipoglycopeptides increasingly used off-label for a variety of Gram-positive infections. While their efficacy has been described in osteomyelitis, bacteremia, and infective endocarditis, evidence specifically addressing cardiovascular prosthetic infections such as prosthetic valve endocarditis (PVE), cardiac implantable electronic device (CIED) infections, left ventricular assist device infections (LVAD), and prosthetic vascular graft infections (PVGI) remains limited. These conditions are particularly challenging due to biofilm formation, difficulties in achieving surgical source control, and the frequent need for prolonged or suppressive therapy. **Objectives**: This systematic review aimed to summarize the available literature on the use of dalbavancin and oritavancin in cardiovascular prosthetic infections, with a focus on therapeutic strategies, clinical outcomes, and safety. **Methods**: We performed a systematic search of PubMed, Embase, Scopus, and Cochrane Library up to 24 June 2025 in accordance with PRISMA guidelines. Eligible studies included adults treated with dalbavancin or oritavancin for cardiovascular prosthetic infections. Data on study characteristics, population demographics, causative pathogens, and microbiological profiles, antibiotic regimens, treatment duration, use of therapeutic drug monitoring (TDM), indication or non-indication for chronic suppressive therapy, adverse events, clinical outcomes, and clinical efficacy were extracted. **Results**: Twenty studies comprising 113 patients were identified, of whom 111 received dalbavancin and 2 oritavancin. The main infections were PVE, CIED, LVAD, and PVGI. Dalbavancin was most effective as consolidation therapy after surgery or device removal, with high cure rates. Prolonged regimens were used as bridging or in partially treated cases, sometimes supported by TDM or PET/CT. Chronic suppressive therapy, mainly for LVAD and PVGI infections, achieved variable outcomes with relapses in about one fifth of patients. Adverse events were infrequent and generally mild. **Conclusions**: The included studies were highly diverse, conducted in various settings and with different objectives. Eight of the twenty included studies were single case reports on dalbavancin and oritavancin, highlighting the predominance of individual case descriptions in the available literature. Long-acting lipoglycopeptides may represent a valuable option for cardiovascular prosthetic infections. Their role appears most favorable as consolidation after adequate source control, while chronic suppressive use showed heterogeneous outcomes. This systematic review was registered on Open Science Framework. This work was supported by grants from the Italian Ministry of Health through Ricerca Corrente, Linea 3, Progetto 3.

## 1. Introduction

Dalbavancin and oritavancin are long-acting parenteral lipoglycopeptide antibiotics derived from teicoplanin. These antibiotics are effective against a wide range of Gram-positive bacteria, including *Staphylococcus* spp., *Streptococcus* spp., and *Enterococcus* spp. [1,2] Dalbavancin and oritavancin have a long elimination half-life and penetrate tissues and biofilms, making them promising options for treating biofilm-mediated infections [3,4,5]. The pharmacokinetic properties and activity profile of dalbavancin and oritavancin support their use for treating cardiovascular prosthetic infections [1,2,3,4,5]. Dalbavancin and oritavancin may represent valuable options also when surgical removal of the prosthesis is not feasible and chronic suppressive therapy is required. In outpatient settings, these antibiotics may reduce hospital stays and improve the quality of life [3,4,5]. Currently, the Food and Drug Administration and the European Medicines Agency approved dalbavancin and oritavancin only for acute bacterial skin and skin structure infections [6]. Although dalbavancin and oritavancin are currently approved only for acute bacterial skin and soft tissue infections, their off-label use in deep-seated Gram-positive infections is increasingly supported by clinical evidence. In the DOTS trial, dalbavancin was non-inferior to vancomycin, daptomycin, or cefazolin in patients with *Staphylococcus aureus* bacteremia and right-sided endocarditis [7]. A randomized study in osteomyelitis confirmed dalbavancin efficacy and safety compared to standard therapy [8]. Retrospective data in osteoarticular infections showed comparable outcomes of dalbavancin compared to vancomycin, daptomycin, and linezolid [9].

Although randomized trials are lacking, observational studies support dalbavancin as consolidation therapy after source control [10,11,12].

For oritavancin, the ongoing ORI-4-CIEDi trial is assessing its role in cardiac device infections caused by multidrug-resistant Gram-positive bacteria [13]. There have been several case series and observational studies highlighting the potential of dalbavancin being used to treat cardiovascular prosthetic infections [14,15]. However, there is limited data on the use of dalbavancin and oritavancin as chronic suppressive therapy for cardiovascular prosthetic infections, such as prosthetic valve endocarditis (PVE), cardiac implantable electronic device (CIED) infections, left ventricular assist device infections (LVAD), and prosthetic vascular graft infections (PVGI) [14,16,17]. Additionally, while recent studies suggest that therapeutic drug monitoring (TDM) could help in the administration of long-acting parenteral lipoglycopeptides in staphylococcal infections, there are scant data on the use of TDM to optimize these antibiotics in patients with cardiovascular prosthetic infections [18,19,20].

We performed a systematic literature review with the aim to evaluate the off-label use of dalbavancin and oritavancin in cardiovascular prosthetic infections and to describe the therapeutic strategies adopted in clinical practice and their clinical outcomes and safety.

## 2. Results

### 2.1. Included Studies

**Figure 1:**
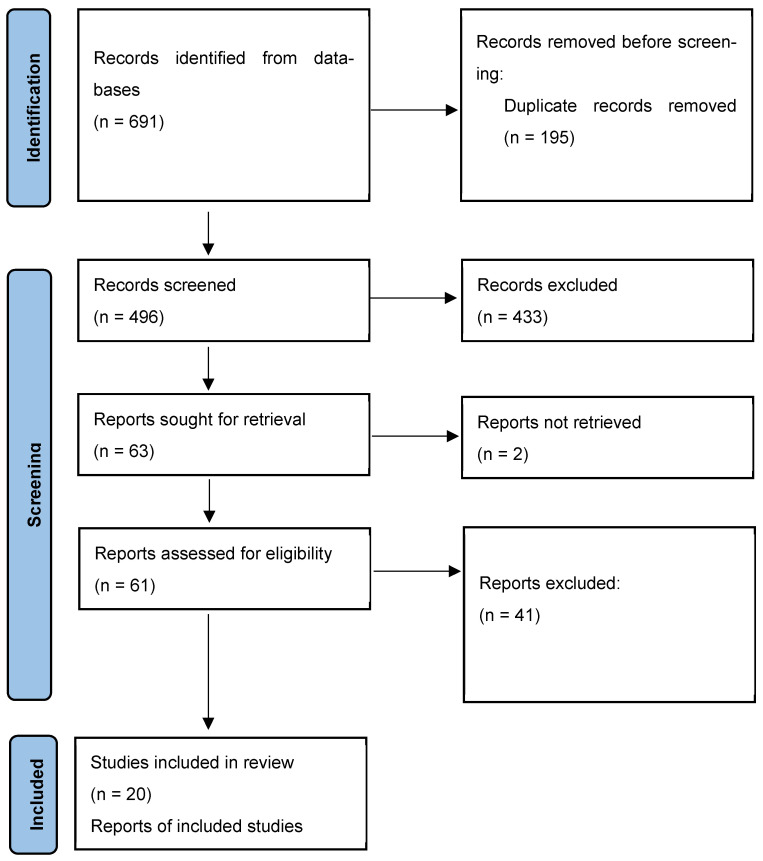
The PRISMA flowchart illustrating the study selection process and the number of the studies included in the systematic review.

The systematic review flow diagram is shown in Figure 1. The initial search yielded 691 articles, of which 195 duplicates were removed. The remaining 496 were screened, leading to the exclusion of 433 studies: 57 due to being background material (e.g., reviews articles, pharmacokinetic/pharmacodynamic studies, regulatory documents), 54 for addressing conditions other than cardiovascular prosthetic infections (e.g., bacteremia, osteomyelitis, orthopedic prostheses, diabetic foot), and 297 for reporting on treatment other than dalbavancin or oritavancin. Additionally, 25 studies were excluded because they lacked clinical outcomes, microbiological data, or dosing information. Sixty-three studies underwent full-text review, but two could not be retrieved. Among the 61 assessed studies, 41 were excluded: 14 reported on native valve endocarditis or lacked sufficient clinical data, 27 studies did not provide data on dalbavancin or oritavancin use in cardiovascular prosthetic infections. Twenty studies met all the inclusion criteria and were included in the final analysis.

### 2.2. Characteristics of the Included Studies

Dalbavancin and oritavancin were evaluated in a total of 20 studies, comprising 18 studies on dalbavancin and 2 on oritavancin [10,12,21,22,23,24,25,26,27,28,29,30,31,32,33,34,35,36,37,38]. The main characteristics are summarized in Table 1 and Table 2 and in Tables S2 and S3. The included studies were conducted in the USA (7 studies), Italy (5 studies), Spain (4 studies), Austria (2 studies), the United Kingdom (1), and Germany (1). The study designs included 8 single case reports, 11 retrospective single- or multicenter case series, and 1 multicenter observational cohort study. The largest cohort was reported in the multicenter Spanish DALBACEN study, which included 23 patients with PVE or CIED infections treated with dalbavancin as consolidation therapy.

### 2.3. Quality of the Studies and Risk of Bias Assessment

The quality appraisal of the studies is reported in Supplementary Table S1. From a qualitative standpoint, the large majority of the included articles fulfilled most of the criteria set by the adopted tools by Murad et al. [39,40,41]. The included studies were evaluated for risk of bias using the Joanna Briggs Institute (JBI) Critical Appraisal Checklist, which is reported in Supplementary Table S4. All studies were considered reliable sources for describing real-life use of long-acting lipoglycopeptide antibiotics in treating cardiovascular prosthetic infections and deemed suitable for inclusion.

### 2.4. Populations of the Included Studies

A total of 20 studies—18 evaluating dalbavancin and 2 oritavancin—accounting for 113 patients were included in the systematic review. The main characteristics of the patients from the included studies are summarized in Table 3. Of the total, 111 patients received dalbavancin and 2 oritavancin.

The mean age was 63.7 years (range 20–89); the patients were 66.1% male and 33.9% female.

The following types of infections were reported among the 111 patients who received dalbavancin: prosthetic valve endocarditis (PVE) (47 cases, 42.3%, including 5 infections on a previous transcatheter aortic valve implantation), CIED infections (22, 19.8%), LVAD infections (23 cases, 20.7%), and prosthetic vascular graft infections (PVGI) (13, 11.7%). In 6 (5.4%) cases infections overlapped—mostly combinations of PVE and VGI or PVE and CIED. The 2 oritavancin cases were PVE and PVGI, respectively.

Microbiological etiology was identified in 109 patients. *S. aureus* and coagulase-negative staphylococci (CoNS) accounted for 60% of isolates, with equal distribution of methicillin-susceptible *S. aureus* (MSSA) and methicillin-resistant *S. aureus* (MRSA). Other identified pathogens included *Enterococcus* spp., *Streptococcus* spp., *Corynebacterium* spp. The cases treated with oritavancin were due to vancomycin-resistant *Enterococcus faecium* and *Staphylococcus lugdunensis*, respectively.

All patients received long-acting lipoglycopeptide antibiotic therapy after the first empirical antibiotic regimen, mostly intravenous (e.g., β-lactams, daptomycin, vancomycin, linezolid), often followed or combined with oral agents (doxycycline, trimethoprim-sulfamethoxazole, tedizolid). Among the 95 cases with available data on the rationale for switching, 72 (63.7%) switched to enable patients to be discharged from hospital, 13 cases (11.4%) due to suboptimal control with oral antibiotics, 6 (5.3%) for toxicity, 1 case due to drug–drug interactions, 1 case due to poor adherence, and 1 case due to a lack of oral options.

### 2.5. Surgical Eligibility and Its Impact on Treatment Strategy

A key factor guiding the therapeutic approach was the presence or absence of surgical operability, which significantly influenced both the choice of strategy and the duration of long-acting antibiotic treatment. Among the 113 patients included, 93 (82.3%) received a formal surgical indication, yet surgery was performed in only 46 (41.6%) patients. In the remaining 47 (41.6%) patients surgery was indicated but not performed due to excessive operative risk, severe comorbidities, or overall clinical frailty. These patients were thus managed with non-curative medical strategies. Operability varied considerably by infection type. In LVAD infections, surgery was indicated in all cases but performed in only 4/23 (17.4%) patients; in PVGI, surgery was performed in only 3/14 (21.4%) cases, with over 75% of patients deemed inoperable. None of the six patients with overlapping prosthetic infections underwent surgery, despite universal indications. By contrast, all 22 (100%) patients with CIED infections who had surgical indication underwent device removal or revision. In PVE 28 (58.3%) patients had a surgical indication, but only 17 (35.4%) underwent surgery, while the remaining 11 (22.9%) were excluded from intervention due to high operative risk. For a detailed breakdown by infection type, see Table 4 and Table 5.

### 2.6. Therapeutic Strategies: Clinical Application of Long-Acting Glycopeptide

To provide a clinically relevant summary of the included studies, we identified three distinct patients’ groups, each corresponding to a specific therapeutic strategy: consolidation therapy, prolonged treatment in partially surgically treated cases, and chronic suppressive therapy. These three categories reflect the definitions previously outlined in the Section 4.

#### 2.6.1. Long-Acting Lipoglycopeptide Antibiotics as Consolidation Therapy in the Treatment of Cardiovascular Prosthetic Infections

All details are summarized in Table 6.

All patients included in this subgroup received dalbavancin as a short-course consolidation strategy; no cases of oritavancin use were reported. Consolidation therapy with dalbavancin was administered to forty-nine patients (43.4%) following completion of a standard intravenous antibiotic regimen.

This subgroup included 32 patients with PVE, including TAVI-associated infections, and 17 with CIED infections. Surgical source control was achieved in 31 cases: all CIED patients underwent device removal, while 14 of the 32 PVE/TAVI patients had valve surgery. The drug was administered as a short-course outpatient therapy, with the goal of completing treatment outside the hospital after adequate initial management. Prior to switching, patients received a median of 3 to 5 weeks of intravenous therapy with agents such as vancomycin, daptomycin, β-lactams like flucloxacillin or cefazolin, fosfomycin, and occasionally rifampicin or gentamicin. Combination therapy during the dalbavancin phase was uncommon, as most patients were treated with monotherapy. Dalbavancin dosing regimens ranged from single-dose administration to one or two weekly or biweekly infusions, with total durations between one and four weeks. Eighteen cases received a 1500 mg single dose. A two-dose regimen consisting of 1000 mg followed by 500 mg one week apart was used in 12 cases. Weekly administration of 1000 mg was documented in 9 patients, while 5 patients received a biweekly regimen of 1500 mg repeated after 14 days. In five additional cases the dosing schedule was not clearly reported.

Adverse events were rare, with one case of nausea [22] and one case of acute renal failure that led to treatment discontinuation [10].

The overall outcome was favorable. Most patients achieved clinical success and microbiological cure. Two cases were classified as clinical failures. One involved a patient in the study by Durante-Magnoni et al. who experienced a relapse of prosthetic valve endocarditis five months after completing dalbavancin, required repeat surgery, and died from postoperative complications [24]. Another patient died from postoperative complications two weeks after valve replacement, shortly after receiving the first dose of dalbavancin [22].

#### 2.6.2. Long-Acting Lipoglycopeptide Antibiotics as Prolonged Therapy in Partially Surgically Treated Cases

All details are summarized in Table 7.

In this subgroup of nineteen patients, long-acting antibiotics were used as prolonged therapy in the context of inoperable infections or incomplete surgical management. Among them, 18 patients received dalbavancin and 1 received oritavancin. The majority had prosthetic valve or TAVI-associated endocarditis (11 cases), followed by CIED infections (4), PVGI (3), and 1 case of LVAD infection. A surgical indication was present in 17 patients, but only 9 underwent partial surgery. The remaining eight patients were judged inoperable due to frailty or comorbidities. In a few cases, long-acting agents were administered after debridement failure or incomplete surgery [25], or to treat residual prosthetic material left in place [24]. In selected cases, dalbavancin served as a bridging strategy before successful heart transplantation [26]. Before switching to long-acting therapy, patients received intravenous antibiotics for a median duration of 4 weeks, with a range spanning from 1 to 104 weeks.

The most frequently used agents prior to the switch included vancomycin, daptomycin, ceftriaxone, flucloxacillin, rifampicin, gentamicin, linezolid, and teicoplanin. The rationale for switching was aimed to enable outpatient treatment in 17/19 cases, whilst 2/19 patients were switched to long-acting lipoglycopeptide for clinical reasons. The most common dalbavancin regimen was 1500 mg every two weeks. Treatment durations ranged from 3 to 35 weeks. Therapeutic drug monitoring (TDM) was applied in three cases [12,28].

One patient received oritavancin [27]. The initial regimen included 1200 mg every other day for three doses, followed by weekly doses for six weeks. After clinical relapse and mitral valve replacement, treatment resumed with 1200 mg twice weekly for ten weeks [27].

No serious or treatment-limiting adverse events were observed across the included cases.

Positron emission tomography/computed tomography (PET/CT) imaging supported management in four cases. In these cases, PET/CT imaging and therapeutic drug monitoring (TDM) were used to guide treatment decisions and assess disease activity, with follow-up extending up to 91 weeks.

The majority of patients achieved both clinical and microbiological cure, even in complex or inoperable cases. Two clinical failures were reported. In one case, a patient with *Staphylococcus aureus* infection relapsed on day 210, suggesting possible reinfection or incomplete eradication [22]. In the other case, the infection evolved from a methicillin-susceptible *S. aureus* (MSSA), initially sensitive to dalbavancin, to a methicillin-resistant strain (MRSA), resistant to dalbavancin [25].

#### 2.6.3. Long-Acting Antibiotics as Chronic Suppressive Therapy for Cardiovascular Infections Involving Non-Removable Prosthetic Material

All details are summarized in Table 8.

A total of 45 patients received long-acting glycopeptides—dalbavancin and in one case oritavancin—as chronic suppressive therapy for persistent cardiovascular infections involving non-removable prosthetic material.

These were patients in which eradication was not feasible due to either surgical contraindications or prior treatment failure. The therapeutic goal was long-term infection control. LVAD infections were the most frequent (*n*: 22), followed by PVGI (*n*: 11), mixed-device infections (*n*: 6), PVE/TAVI (*n*: 5), and CIED infection (*n*: 1). Before switching to long-acting agents, patients received prior antibiotic therapy for a median of 13.5 weeks (mean 24.2, range 1–140), commonly vancomycin, daptomycin, linezolid, rifampicin, or ceftriaxone. Outpatient parenteral antibiotic therapy (OPAT) was often a motivation, but switching to long-acting agents also occurred because of clinical failure, toxicity, drug interactions, poor adherence, or absence of effective oral alternatives.

Treatment durations ranged from 12 to >124 weeks, with a median of 13 infusions.

Therapeutic drug monitoring (TDM) was employed in eight patients to individualize the dosing interval of dalbavancin. TDM was used to ensure drug exposure remained adequate over time. In one case of PVG, TDM was specifically used to prevent underexposure, allowing for dose adjustment based on drug levels to ensure sustained efficacy during chronic suppressive therapy [12].

In eight cases, [^18^F]-FDG PET/CT played a central role in treatment monitoring and decision-making. In one case, PET/CT confirmed metabolic improvement, supporting over six months of therapy [31]. In Gallerani et al. PET/CT was performed in 9/12 patients at 6 months, with 6 patients showing reduced uptake [12]. In another case, PET/CT at nearly one year revealed persistent LVAD infection, leading to continuation of suppressive therapy [29].

Overall, adverse events were infrequent and generally mild, occurring in 4/45 (8.8%) patients. These included a mild rash, asthenia, and transient renal impairment. Only one case required permanent discontinuation of dalbavancin due to liver toxicity [32].

Among the patients, 16/45 (35.5%) achieved stable infection suppression and discontinued dalbavancin switching to oral regimens (e.g., doxycycline, minocycline), guided by clinical stability, PET/CT findings, or patient preference. One patient stopped dalbavancin after heart transplantation, which enabled definitive source control through LVAD removal.

Conversely, 9/45 patients (20%) experienced therapeutic failure, mainly due to uncontrolled infection with breakthrough bacteremia or relapse, prompting a switch to alternative antibiotics (e.g., daptomycin, ceftaroline, vancomycin) and hospital readmission. LVAD replacement was eventually required in at least three patients, either due to persistent infection or mechanical complications.

Four additional cases were labeled as having “good clinical response” but lacked clear documentation of treatment completion and were therefore considered as uncertain outcomes [32].

### 2.7. Adverse Events and Safety

Overall, dalbavancin was well tolerated. Among the 111 patients treated with dalbavancin, 14 (12.6%) experienced adverse events. Most reported adverse effects were mild to moderate and did not require treatment discontinuation. However, two patients (1.8%) had to discontinue therapy due to renal impairment [10] and combined renal and hepatic toxicity [32]. The most frequently observed adverse events included skin reactions rash, 3 patients [12,24,29], thrombocytopenia, 3 patients [24], transient elevation of liver enzymes, 2 patients [24], and gastrointestinal symptoms (1 patient with nausea) [22].

## 3. Discussion

The aim of this systematic review was to summarize the emerging role of long-acting glycopeptides in managing prosthetic cardiovascular infections.

Overall, a total of 113 patients were identified from the 20 included studies. Among them, 93 (83.8%) achieved clinical success, which was defined as microbial eradication and no evidence of relapse of infection. These findings highlight the promising potential of long-acting glycopeptide antibiotics in managing prosthetic cardiovascular infections.

So far, several published reports have examined the effectiveness of dalbavancin for treating infective endocarditis, particularly in sequential treatment plans after initial intravenous therapy. A recent literature synthesis encompassing multiple case series and reports estimated clinical success rates at around 75–80%, suggesting consistent performance across diverse Gram-positive etiologies. Notably, a large multicenter cohort study conducted in Spain reported favorable outcomes in over 90% of cases at the 12-month follow-up [17]. However, the majority of patients had undergone surgical intervention prior to dalbavancin initiation and a significant proportion was treated for native valve endocarditis rather than prosthetic infections [42]. Therefore, our finding of a total 83.8% clinical success rate confirms the potential of using long-acting glycopeptide antibiotics in managing patients with cardiovascular prosthetic infections.

In our systematic review, among the 18 patients (16.2%) who experienced treatment failure, this was primarily due to persistent or breakthrough infections, microbiological relapse—especially in PVE and PVGI—or emergence of antimicrobial resistance, which was documented in at least 3 patients. Overall, 9 deaths were reported, 6 of which were infection-related and 3 due to non-infectious causes. Treatment failure was mostly observed in patients with non-removable prosthetic material, not eligible for full surgical intervention and source control. These data underscore the clinical complexity of this patient population, which is often ineligible for surgical intervention and is instead managed with long-term, non-curative strategies.

According to the 2023 ESC guidelines, surgery is recommended in complicated PVE, while conservative treatment may be considered in selected cases [43]. Many patients are ineligible for surgery due to age or comorbidities, leading to worse outcomes [43]. In such scenarios, long-acting glycopeptides may offer a practical and well-tolerated alternative for chronic suppressive therapy. Their favorable PK/PD properties allow for widely spaced dosing, reducing the need for prolonged hospitalization and facilitating outpatient management. These features are especially valuable in patients for whom hospital-based therapy is not feasible or desirable [44]. However, our findings suggest that the outcome of patients’ cardiovascular prosthetic infections who have not undergone adequate source control remains poor, even with the long-term administration of long-acting glycopeptide antibiotics.

This review has several important limitations. Firstly, the included studies were highly diverse, conducted in various settings and with different objectives. Eight included studies were single case reports, limiting generalization. Heterogeneity in study designs and monitoring strategies (e.g., PET/CT, TDM) further limited comparability, requiring cautious interpretation of findings.

Notably, we observed significant heterogeneity in the reported patient populations, with surgical removal being more prevalent in PVE and CIED infections, whereas chronic suppressive therapy was predominantly used in patients with LVAD and PVGI infections where surgical intervention was not feasible. Patients from the different included studies involved those with a documented indication for chronic suppressive therapy, those treated for the removal of infected prostheses, and cases of intermediate indication where the necessity for long-term therapy is debatable.

Given the significant heterogeneity among patients in terms of infection type, surgical eligibility, and the intended purpose of therapy, we divided the cohort into three distinct groups to provide a clearer, more clinically relevant overview. These groups correspond to specific therapeutic strategies: consolidation therapy, prolonged treatment in partially treated cases or patients awaiting surgery, and chronic suppressive therapy.

The classification into three therapeutic strategies—consolidation, prolonged, and chronic suppressive therapy—effectively reflected clinical intent and complexity.

Notably, consolidation therapy involving long-acting glycopeptides was successful in the forty-nine patients who underwent adequate source control, with few adverse events reported.

Conversely, among the 45 patients from the included studies who received long-acting antibiotics as chronic suppressive therapy, only 16 (35.5%) achieved prolonged, stable infection suppression, while 9 (20%) experienced therapeutic failure, primarily due to uncontrolled infection with breakthrough bacteremia or relapse. This prompted a switch to alternative antibiotics (e.g., daptomycin, ceftaroline, or vancomycin) and hospital readmission. In this patient group, the mortality rate remained high even with chronic suppressive treatment involving long-acting glycopeptides. In our opinion, these data confirm the limitations of chronic antibiotic regimens in patients who are unable to undergo surgical source control.

Regarding the use of radiological examinations, such as PET/CT, to inform treatment decisions for patients for whom device removal is not an option, the studies included in this review mostly relied on clinical follow-up. PET/CT imaging was used in only a few cases to determine treatment duration. The limited and non-standardized use of PET/CT imaging reduced consistency across studies, making it impossible to draw conclusions about its usefulness in informing therapeutic decisions for these patients [37,45].

Interestingly, therapeutic drug monitoring was reported in eight patients and was employed to customize dalbavancin dosing during long-term therapy, particularly in PVE and PVGI cases. Most studies adopted an initial regimen of 1500 mg on days 1 and 8, followed by variable dosing intervals—often 1000 mg every two weeks or individualized schedules based on TDM—to maintain plasma concentrations above conservative efficacy thresholds (≥4.02–8.04 mg/L) [1,12,18]. In the chronic suppressive therapy scenario, such a TDM-guided approach may represent a valuable strategy to ensure adequate drug exposure over time, potentially preventing complications and improving patient outcomes [18,46].

Of the studies included in our systematic review, only two cases involved the use of oritavancin. Of these, only one was consistent with suppressive intent, involving 16 weekly infusions without therapeutic drug monitoring and limited follow-up [30]. In the other case, oritavancin was administered perioperatively in a patient with PVE due to Vancomycin-resistant *Enterococcus*, with subsequent relapse and valve replacement [27]. While both cases suggest the potential for clinical use, the data are insufficient to draw conclusions.

## 4. Materials and Methods

This systematic review was conducted in accordance with the Preferred Reporting Items for Systematic Reviews and Meta-Analyses (PRISMA) guidelines. The study was registered on the Open Science Framework (OSF; DOI: https://doi.org/10.17605/OSF.IO/GJUEZ).

### 4.1. Systematic Search and Libraries

To ensure a structured selection process, articles were screened using Rayyan version 1.4.3, a web-based platform for systematic reviews, while data extraction was performed using Excel spreadsheets, version 2.76, for further analysis. A comprehensive search was conducted in PubMed, Embase, Cochrane Library, and Scopus. The final search was completed on 24 June 2025. The search strategy included keywords related to long-acting antibiotics, cardiovascular prosthetic infections, and chronic suppressive therapy, without applying filters, language restrictions, or time limits.

### 4.2. Eligibility Study Criteria and Work Process

The following inclusion criteria were applied:

(i) randomized controlled trials, non-randomized intervention studies, observational studies including single case or case series reporting clinical outcomes, microbiological data, or antibiotic dosing regimens of dalbavancin or oritavancin treatment for cardiovascular prosthetic infections;

The following exclusion criteria were applied:

(i) preclinical studies, in vitro studies, or studies in the animal model;
(ii) studies not reporting on infections involving cardiovascular prostheses, e.g., bacteremia, osteomyelitis, native valve endocarditis
(iii) unpublished studies, preprints, conference abstracts, or studies published without a peer-review process.

Duplicate records were manually identified and removed. Once the eligible studies were identified, data regarding study characteristics, population demographics, causative pathogens and microbiological profiles, antimicrobial sensitivity to dalbavancin and oritavancin, antibiotic regimens, treatment duration, use of TDM, indication or non-indication for chronic suppressive therapy, adverse events, clinical outcomes, and clinical efficacy as defined by each individual study were extracted and collected.

### 4.3. Classification of Therapeutic Strategies

To enable a systematic assessment of clinical practices, treatment strategies involving long-acting glycopeptides (dalbavancin or oritavancin) were retrospectively categorized into three predefined groups based on therapeutic intent, treatment duration, surgical eligibility, and underlying clinical context, as follows:

#### 4.3.1. Consolidation Therapy

Definition: Short-term sequential treatment with a long-acting glycopeptide following initial standard intravenous (IV) antibiotic therapy.

Clinical context: This strategy was employed in patients who had already received a course of adequate antibiotic therapy, often combined with surgical procedures or device removal, and in whom dalbavancin or oritavancin was administered to complete the antibiotic course. The primary objectives included minimizing hospitalization duration, facilitating outpatient care (e.g., outpatient parenteral antibiotic therapy, OPAT), and ensuring treatment continuity.

#### 4.3.2. Prolonged Therapy in Partially Surgically Treated Cases

Definition: Medium- to long-term treatment with dalbavancin in patients with prosthetic valve endocarditis (PVE) or similar conditions who were not eligible for full surgical intervention.

Clinical context: This approach was adopted in patients with a formal indication for surgery who could not immediately undergo the procedure due to prohibitive surgical risk, advanced age, or comorbidities. It also encompassed cases where patients had undergone partial surgical management (e.g., drainage, debridement) or were receiving long-acting therapy as a bridging solution while awaiting delayed or staged interventions. Treatment was typically prolonged but with a defined endpoint.

#### 4.3.3. Chronic Suppressive Therapy

Definition: Indefinite or long-term suppressive use of a long-acting glycopeptide in cardiovascular prosthetic infections patients with non-removable prosthetic material.

Clinical context: This strategy was applied to patients with persistent or relapsing infections for whom neither curative surgery nor full eradication of the infection was feasible. Dalbavancin or oritavancin was used with the intent to suppress microbial activity, prevent clinical progression, and reduce the risk of relapse in a palliative or containment-oriented framework.

This classification framework was developed post hoc during data extraction and analysis to facilitate a consistent comparison of clinical scenarios, treatment regimens, and outcomes across the included studies. It also guided the organization of the Section 2 and Section 3.

### 4.4. Definitions Adopted in the Study

Clinical success: According to the 2023 ESC guidelines, clinical success was defined as documented microbial eradication and no evidence of relapse of infection [46].

Chronic suppressive therapy: Antimicrobial therapy for an undetermined duration, intended to prevent relapse of the infection.

Therapeutic failure: The occurrence of uncontrolled infection after the start of proper antibiotic treatment, including breakthrough bacteremia and occurrence of relapse.

Relapse: According to the 2023 ESC guidelines, a repeated episode of infective endocarditis was caused by the same microorganism. Relapse represents a therapeutic failure due to insufficient duration of initial treatment, sub-optimal choice of initial antibiotics, or a persistent focus of infection [43].

Breakthrough bacteremia: an episode of continuous or new-onset bacteremia in a patient receiving appropriate antibiotics for the microorganism recovered from blood cultures.

Microbial eradication: Microbial eradication is assumed when blood cultures become and remain negative after initiation of appropriate antimicrobial therapy. If valve or prosthetic material is removed surgically, negative cultures from excised tissue support microbial eradication.

### 4.5. Quality of the Included Studies and Risk of Bias Assessment

The quality of the included studies was appraised autonomously by two reviewers (FG and GG), and any potential discrepancy was resolved through consensus of the entire study group [39]. To evaluate the quality of case reports and case series, an adapted version of the tool proposed by Murad et al. was implemented, investigating the following domains (through eight items): selection, ascertainment, causality, and reporting [40]. Quality appraisal was based on the overall study, regardless of the number of cases extracted. Since summary quality scores may generate misleading results, an overall judgment on the methodological quality of included studies was deemed more appropriate [40,41].

Considering the inherited limitations of case series and case reports, which lack control groups and are prone to selection and publication bias. Considering that most included studies were retrospective, increasing the risk of information bias and confounding due to incomplete or selective data reporting, an evaluation of the risk of bias has been performed. As standard tools like ROBINS-I or Newcastle–Ottawa Scale are not suited for the case series and case reports study designs, to evaluate the risk of bias of the included studies we used adapted criteria from the Joanna Briggs Institute (JBI) Critical Appraisal Checklist.

### 4.6. Synthesis of Findings

Given the heterogeneity of the included studies, a meta-analysis was deemed unfeasible. Instead, a systematic narrative synthesis was conducted, summarizing study characteristics in tables and discussing the overall level of evidence.

Given the marked heterogeneity of the patient population in terms of infection type, surgical eligibility, and clinical intent of therapy, a simple pooled analysis would have failed to capture the complexity of the therapeutic approaches. To provide a more clinically relevant overview, we divided the cohort into the following three distinct groups, each corresponding to a specific therapeutic strategy: consolidation therapy, prolonged treatment in inoperable or partially treated cases, and chronic suppressive therapy. This predefined classification allows for a structured comparison of treatment contexts, durations, and drug utilization patterns, thereby facilitating interpretation despite the variability in patient management. Each subgroup has been described separately, with a specific focus on the role of dalbavancin and oritavancin within each therapeutic context.

## 5. Conclusions

Available evidence suggests that long-acting glycopeptides, i.e., dalbavancin, may offer a valuable therapeutic option in selected cases of cardiovascular prosthetic infections. Clinical success was most consistently reported in patients with adequate source control or when long acting was used as part of a consolidation strategy. In suppressive therapy settings, outcomes were more heterogeneous. Patients with cardiovascular prosthetic infections for whom surgical intervention and source control was not feasible had a higher mortality rate, even with chronic suppressive treatment involving long-acting glycopeptides. While TDM and PET/CT may help personalize management, further prospective studies are needed to define optimal treatment strategies.

## Figures and Tables

**Table 1:** Studies by country.

Country	No. of Studies	Study Types
USA	7	1 retrospective multicenter study; 4 retrospective case series, 2 case reports
Italy	5	4 retrospective case series, 1 case report
Spain	4	2 retrospective case series, 1 multicenter cohort, 1 case report
Austria	2	1 retrospective case series, 1 case report
UK	1	1 retrospective case series
Germany	1	1 retrospective case series

**Table 2:** Summary of the studies included in the systematic review.

Reference	Study Type	Infection Focus in the Study	Main Treatment Approach	No. of Patients
Tobudic et al., 2018 [22]	Single-center retrospective study (Austria)	PVE, CIED	Consolidation therapy with dalbavancin	11
Kussmann et al., 2018 [25]	Single case report (Austria)	CIED	Suppressive therapy with dalbavancin	1
Spaziante et al., 2019 [31]	Single case report (Italy)	PVE, CIED	Suppressive therapy with dalbavancin	1
Morrisette et al., 2019 [36]	Retrospective multicenter study (USA)	LVAD	Suppressive therapy with dalbavancin	1
Hidalgo-Tenorio et al., 2019 [10]	Multicenter, observational, retrospective study (Spain)	PVE, CIED	Consolidation therapy with dalbavancin	23
Howard-Anderson et al., 2019 [37]	Single case report (USA)	LVAD	Suppressive therapy with dalbavancin	1
Deida et al., 2020 [38]	Single-center retrospective case series (USA)	PVE, CIED	Suppressive therapy with dalbavancin	2
Hitzenbichler et al., 2020 [29]	Single-center retrospective case series (Germany)	OVE, LVAD, TAVI, PVGI	Suppressive therapy with dalbavancin	4
Ciccullo et al., 2020 [28]	Single-center retrospective study (Italy)	PVGI	Suppressive therapy with dalbavancin	1
Durante-Magnoni et al., 2021 [24]	Single-center retrospective study (Italy)	PVE, TAVI, CIED	Consolidation therapy with dalbavancin	7
Guleri et al., 2021 [21]	Single-center retrospective case series (UK)	PVGI	Suppressive therapy with dalbavancin	4
Ruiz-Sancho et al., 2023 [32]	Multicenter retrospective study (Spain)	PVGI	Suppressive therapy with dalbavancin	6
Rowe et al., 2023 [35]	Single-center retrospective case series (USA)	LVAD	Suppressive therapy with dalbavancin	8
Gallerani et al., 2023 [12]	Single-center retrospective case series (Italy)	PVE, CIED, PVGI	Suppressive therapy with dalbavancin	14
Mansoor et al., 2023 [26]	Single-center retrospective case series (USA)	LVAD	Suppressive therapy	10
Pallotto et al., 2023 [34]	Single-center retrospective case series (Italy)	LVA, PVGI, PVE	Suppressive therapy with dalbavancin	4
Salinas-Botrán et al., 2024 [23]	Single-center retrospective case series (Spain)	PVE, CIED, PVGI	Consolidation therapy with dalbavancin	11
Cepeda et al., 2024 [33]	Single case report (Spain)	TAVI	Suppressive therapy with dalbavancin	1
Johnson et al., 2015 [27]	Single case report (USA)	PVE	Prolonged therapy with oritavancin	1
Schulz et al., 2017 [30]	Single-center retrospective case series (USA)	PVGI	Suppressive therapy with oritavancin	1

PVE: prosthetic valve endocarditis; CIED: cardiac implantable electronic device infections; LVAD: left ventricular assist device infections; PVGI: prosthetic vascular graft infections; IE: infective endocarditis.

**Table 3:** Summary of the patient characteristics from the included studies.

Category	Value
Total studies included	20
Dalbavancin studies	18
Oritavancin studies	2
Total patients	113
Dalbavancin-treated	111
Oritavancin-treated	2
Mean age (*n* = 74)	63.7 years (20–89)
Sex distribution (*n* = 59)	
Male	39 (66.1%)
Female	20 (33.9%)
Type of prothesis	
PVE/TAVI	48 (42.5%)
CIED	22 (19.5%)
LVAD	23 (20.4%)
PVGI	14 (12.4%)
Overlapping infections	6 (5.3%)
Microbiology	
*S. aureus* (MSSA/MRSA)	36 (32.4%)
CoNS (incl. *S. epidermidis*)	31 (27.4%)
*Enterococcus* spp.	14 (12.4%)
*Streptococcus* spp.	12 (10.6%)
*Corynebacterium* spp.	8 (7.1%)
Polymicrobial	9 (8%)
*Gemella morbillorum*	1 (0.9%)
Unknown etiology	2 (1.8%)
Reason for switching to long-acting therapy (*n* = 95)	
OPAT/early discharge	72 (63.7%)
Oral therapy failure	13 (11.4%)
Adverse events/toxicity	6 (5.3%)
Drug interactions	1 (0.9%)
Poor adherence	1 (0.9%)
No oral option available	1 (0.9%)

PVE: prosthetic valve endocarditis; CIED: cardiac implantable electronic device infections; LVAD: left ventricular assist device infections; PVGI: prosthetic vascular graft infections; IE: infective endocarditis; MSSA: meticillin-sensitive *Staphylococcus aureus*; MRSA: meticillin-resistant *Staphylococcus aureus*; CoNS: coagulase-negative *Staphylococci*; OPAT: outpatient parenteral antimicrobial therapy.

**Table 4:** Surgical indication by type of infection.

Infection Type	N with No Surgical Indication	N with Surgery Indication	Surgery Performed	Surgery Not Performed Due to High Surgical Risk
Total 113	20 (17.7%)	93 (82.3%)	46 (41.6%)	47 (41.6%)
PVE 48	20 (41.7%)	28 (58.3%)	17 (35.4%)	11 (22.9%)
CIED 22	0	22 (100%)	22 (100%)	0
LVAD 23	0	23 (100%)	4 (17.4%)	19 (82.6%)
PVGI 14	0	14 (100%)	3 (21.4%)	11 (78.6%)
Overlap 6	0	6 (100%)	0	6 (100%)

PVE: prosthetic valve endocarditis; CIED: cardiac implantable electronic device infections; LVAD: left ventricular assist device infections; PVGI: prosthetic vascular graft infections.

**Table 5:** Therapeutic strategies and main outcome of the patients with cardiovascular prosthetic infections treated with dalbavancin or oritavancin.

Therapeutic Strategy	Patients (*n*, %)	Type of Prothesis	Surgical Intervention (*n*, %)	Duration of Therapy	Long Acting Used	TDM/PET/CT Use	Key Findings
Consolidation Therapy	49/113 (43.4%)	PVE/TAVI 32; CIED 17	31/49 patients underwent surgery	1–4 weeks	Dalbavancin	No	Short-term use post-standard IV therapy to complete treatment, mostly after the removal of the infected device or source control surgery. High rate of clinical success, higher than 90%.
Prolonged Therapy in Inoperable PVE or Partially Managed Surgical Cases.	19/113 (16.8%)	11 PVE/TAVI 4 CIED, 1 LVAD, 3 PVGI	9/19 underwent surgery; the remaining 8 had surgical indications but were considered high-risk	5–35 weeks	Dalbavancin, oritanvancin	TDM in 3 studies; PET7TC in 4 studies	Mixed cases: some had surgical indications but were deemed too high risk; others underwent staged or partial procedures either before or after long-acting therapy, which was occasionally used as a bridge to surgery. Clinical success rate 80–90%
Chronic Suppressive Therapy for Persistent or Non-removable Infections	45/113 (39.8%)	5 PVE/TAVI 1 CIED, 22 LVAD, 11 PVGI, 6 combinations	6/45 underwent partial procedures or surgery following therapeutic failure	Months to >2 years	Dalbavancin or oritavancin	Yes (TDM in 8 studies, PET/CT in 8 selected cases)	Suppressive therapy of indefinite or prolonged duration for persistent prosthetic infections—either non-removable or partially managed—including post-surgical relapses. Duration varied widely; treatment was still ongoing in 16 of 45 cases

PVE: prosthetic valve endocarditis; CIED: cardiac implantable electronic device infections; LVAD: left ventricular assist device infections; PVGI: prosthetic vascular graft infections; TAVI: transcatheter aortic valve implantation; TDM: therapeutic drug monitoring, IV: intravenous.

**Table 6:** Characteristics of the patients with cardiovascular prosthetic infections treated with long-acting lipoglycopeptide antibiotics as consolidation therapy—summary table.

Consolidation Therapy	Details
Total Patients	49 patients
Long-Acting Agent	Dalbavancin (100%)
Infection Type	32 PVE/TAVI, 17 CIED
Surgical Intervention	Surgery in 14/32 PVE (43.8%), device removal in 17/17 CIED (100%)
Main Pathogens	*S. epidermidis*, MSSA, MRSA, *Enterococci* spp., *Streptococci* spp., CoNS
Prior IV Antibiotics (Weeks)	Median 3–5 weeks
Common IV Agents Used	Vancomycin, Daptomycin, Fosfomycin, Rifampicin, β-lactams (flucloxacillin, cefazolin), Gentamicin
Dalbavancin Regimen	Single-dose or 1–2 weekly/biweekly infusions
Duration of Dalbavancin Therapy	1–4 weeks
Dalbavancin Regimens	1500 mg single dose (18 patients), 1000 + 500 mg (12 patients), 1000 mg weekly (9 patients), 1500 mg biweekly (5 patients), other/unknown (5 patients)
Combination Therapy	Monotherapy in majority, occasional use of concurrent oral agents
Therapeutic Drug Monitoring	Not used
PET/CT Imaging	Not used
Adverse Events	1 patient had nausea, 1 acute kidney injury
Clinical Failures	1 death due to post-surgery complications, 1 relapse with later death, 2 treatment failures requiring valve replacement
Still on Therapy at Follow-up	None
Clinical Success Rate	High (>90%)
Follow-up Duration	2 to 12 months

PVE: prosthetic valve endocarditis; CIED: cardiac implantable electronic device infections; MSSA: meticillin-sensitive *Staphylococcus aureus*; MRSA: meticillin-resistant *Staphylococcus aureus*; CoNS: coagulase-negative *Staphylococci,* IV: intravenous.

**Table 7:** Characteristics of the patients with cardiovascular prosthetic infections treated with long-acting lipoglycopeptide antibiotics as prolonged therapy.

Prolonged Therapy	Details
Total Patients	19 patients (16.8%)
Long-Acting Agent	Dalbavancin (*n* = 18), Oritavancin (*n* = 1)
Infection Type	11 PVE/TAVI, 4 CIED, 3 PVGI, 1 LVAD
Surgical Intervention	Surgery not performed or incomplete in all; partial procedures in some
Main Pathogens	MSSA, MRSA, *Enterococcus faecalis*, CoNS, Gemella, *Streptococcus* spp.
Prior IV Antibiotics (Weeks)	Median 4 weeks (range: 1–104 weeks)
Common IV Agents Used	Vancomycin, Daptomycin, Ceftriaxone, Flucloxacillin, Rifampicin, Gentamicin, Linezolid, Teicoplanin
Dalbavancin Regimen	Weekly or biweekly
Duration of Dalbavancin Therapy	3–35 weeks (mostly 4–10 weeks)
Dalbavancin Regimens	1500 mg biweekly (11 pts), 1000 + 500 mg weekly (6 pts), other (2 pts)
Combination Therapy	Monotherapy
Therapeutic Drug Monitoring	Used in 3 patients [12,28]
PET/CT Imaging	Used in selected cases to guide duration [12,28]
Adverse Events	1 rash, 1 acute kidney injury and skin reaction (both mild and resolved)
Clinical Failures	2 (1 relapse day 210, 1 resistance emergence from MSSA→MRSA)
Still on Therapy at Follow-up	All completed therapy; 1 bridge to transplant
Clinical Success Rate	High
Follow-up Duration	Up to 91 weeks (oritavancin: 22 months)

PVE: prosthetic valve endocarditis; CIED: cardiac implantable electronic device infections; LVAD: left ventricular assist device infections; PVGI: prosthetic vascular graft infections; MSSA: meticillin-sensitive *Staphylococcus aureus*; MRSA: meticillin-resistant *Staphylococcus aureus*; CoNS: coagulase-negative *Staphylococci*.

**Table 8:** Characteristics of the patients with cardiovascular prosthetic infections treated with long-acting lipoglycopeptide antibiotics as chronic suppressive therapy.

Chronic Suppressive Therapy	Details
Total Patients	45 patients (40.5%)
Long-Acting Agent	Dalbavancin (*n* = 44), Oritavancin (*n* = 1)
Infection Type	22 LVAD, 11 PVGI, 6 mixed-device, 5 PVE/TAVI, 1 CIED
Surgical Intervention	Surgery not feasible due to contraindications or prior failure
Main Pathogens	*S. aureus* (MSSA/MRSA), *E. faecalis,* CoNS, *Streptococcus* spp.
Prior IV Antibiotics (Weeks)	Median 13.5 weeks (mean 24.2; range: 1–140 weeks)
Common IV Agents Used	Vancomycin, Daptomycin, Linezolid, Rifampicin, Ceftriaxone
Dalbavancin Regimen	Weekly or biweekly, or TDM-guided dosing
Duration of Dalbavancin Therapy	12 to >124 weeks (median 13 infusions; range 2–175)
Dalbavancin Regimens	1500 mg biweekly (most), 1000 + 500 mg weekly, 375 mg weekly in 1 patient (renal impairment)
Combination Therapy	Monotherapy or sequential oral switch (e.g., doxycycline)
Therapeutic Drug Monitoring	Used in 8 patients
PET/CT Imaging	Used in at least 8 patients; guided duration in several cases
Adverse Events	4 mild events: rash, renal impairment, asthenia, hepatic toxicity (1 discontinuation)
Clinical Failures	9 failures (20%): persistent infection, relapse, resistance (1 case), 2 deaths
Still on Therapy at Follow-up	16 still on therapy at follow-up (35.5%)
Clinical Success Rate	Clinical success rate < 80%, lower than for consolidation or prolonged therapy. Infection control in many patients but with clinical uncertainty
Follow-up Duration	Limited post-therapy data

PVE: prosthetic valve endocarditis; CIED: cardiac implantable electronic device infections; LVAD: left ventricular assist device infections; PVGI: prosthetic vascular graft infections; MSSA: meticillin-sensitive *Staphylococcus aureus*; MRSA: meticillin-resistant *Staphylococcus aureus*; CoNS: coagulase-negative *Staphylococci*; TDM: therapeutic drug monitoring, IV: intravenous.

## Data Availability

The original data presented in the study are openly available online in PubMed, Embase, Scopus, and Cochrane Library.

## Supplementary Materials

The following supporting information can be downloaded at: https://www.mdpi.com/article/10.3390/antibiotics14111130/s1, Table S1. Quality appraisal of case reports and case series according to the tool proposed by Murad et al. [40], presented here in a modified version. Table S2. Cohort studies included in the review on patients treated with dalbavancin for cardiovascular prosthetic infections. Table S3. Studies included in the review on patients treated with oritavancin for cardiovascular prosthetic infections. Table S4. Risk of bias appraisal of the included studies using the Joanna Briggs Institute (JBI) Critical Appraisal Checklist.
