# Supplementary material for: Long-Term Therapy with Long-Acting Lipoglycopeptide Antibiotics in the Treatment of Cardiovascular Prosthetic Infections: A Systematic Review
Source: Antibiotics (Basel). 2025 Nov 7;14(11):1130. doi: 10.3390/antibiotics14111130 (PMC12649424; doi:10.3390/antibiotics14111130)
Supplement: Supplementary file 1 [file antibiotics-14-01130-s001.zip › antibiotics-3931926-supplementary.pdf]

**Table S1.** Quality appraisal of case reports and case series according to the tool proposed by Murad et al. [40], presented here in a modified version [a].

| Study | Selection | Ascertainment | | Causality | | Reporting |
|---|---|---|---|---|---|---|
| | **Does the patient(s) represent(s) the whole experience of the investigator (centre) or is the selection method unclear to the extent that other patients with similar presentation may not have been reported?** | **Was the exposure adequately ascertained?** | **Was the outcome adequately ascertained?** | **Were other alternative causes that may explain the observation ruled out?** | **Was follow-up long enough for outcomes to occur?** | **Is the case(s) described with sufficient detail to allow other investigators to replicate the research or to allow practitioners to make inferences related to their own practice?** |
| Morrisette [36] | * | * | * | * | * | * |
| Howard-Anderson [37] | * | * | * | * | * | * |
| Kussmann [25] | | * | * | * | * | * |
| Tobudic [22] | * | * | * | * | * | * |
| Salinas-Botrána [23] | * | * | * | * | * | * |
| Guleri [21] | | * | * | * | * | * |
| Johnson [27] | | * | * | * | * | * |
| Cepeda [33] | | * | * | * | * | * |
| Pallotto[34] | * | * | * | * | * | * |
| Mansoor [26] | * | * | * | * | * | |
| Gallerani [12] | | * | * | * | * | * |
| Rowe [35] | * | * | * | * | * | * |
| Ruiz-Sancho [32] | * | * | * | * | * | * |
| Durante-Mangoni, [24] | * | * | * | * | * | * |
| Ciccullo [28] | | * | * | | * | * |
| Hitzenbichler [29] | * | * | * | * | * | * |
| Spaziante [31] | * | * | * | * | * | * |
| Deida [38] | * | * | * | * | * | * |
| Hidalgo-Tenorio [10] | * | * | * | * | * | * |
| Schulz [30] | | * | * | * | * | |

[a] For the purposes of the present review, two of the original eight items were considered as ‘not applicable’ (namely, ‘Was there a challenge/rechallenge phenomenon?’ and ‘Was there a dose–response effect?’) since they are mostly relevant to cases whose primary aim is to investigate adverse drug events.

**Table S2.** Cohort studies included in the review on patients treated with dalbavancin for cardiovascular prosthetic infections.

| | Study type | Reference | No. of patients | Organism(s) | Type of Infection | Prosthetic removal | Indication for suppressive antibiotic therapy | Antibiotics used prior to dalbavancin | Dalbavancin dose | Thera peutic drug monit oring | Dalbavancin duration | Adverse events/ failure of dalbavancin | Clinical Success during treatment | Follow-Up (Weeks) |
|---|---|---|---|---|---|---|---|---|---|---|---|---|---|---|
| 1 | Single-centre retrospective study (Austria) | Tobudic et al, 2018 [22] | 11 | *Staphylococcus aureus* - 3 *Streptococcus* spp. -3 Coagulase-negative *Staphylococci* - 2 *Enterococcus faecalis* - 4 | PVE 6, CIED 5 | 1 of 6 with PVE 4 of 5 with CIED | Not specified | Various antibiotic treatment | 1500 mg followed by 1000 mg or 1000 mg followed by 500 mg: Once or twice weekly | no | 1 to >30 weeks | One patient had nausea and vomiting but continued therapy. Another developed a 2.5-fold creatinine increase after two weeks, which normalized after dose reduction. | Clinical success in 81% of the patients. In 1 case a dalbavancin non-susceptible isolate emerged. 1 *E. faecalis* PVE patient switched to dalbavancin died from post-surgical complications, classifying the case as a failure | Not specified |
| 2 | Single case report (Austria) | Kussmann et al, 2018 [25] | 1 | MSSA | CIED | No | Yes | Various antibiotic treatment | Not specified | no | Approximately 4 months in combination with rifampicin and approximately 2 months as monotherapy | No | Therapeutic failure. Two initial unsuccessful attempts of pacemaker explant. Delayed pacemaker explant. 11 months after presentation a dalbavancin non-susceptible isolate emerged | |
| 3 | Single case report Suppressive therapy (Italy) | Spaziante et al, 2019 [31] | 1 | *Staphylococcus epidermidis* | PVE | No | Yes | Daptomycin plus cetriaxone for 7 weeks | Dalbavancin (1500-mg dose) was administered when serum bactericidal activity titers fell to ≤1:8 | Yes | Received five doses of 1500 mg over a period of 189 days (27 weeks) ongoing | No | Clinical success, no recurrence, minimal PET-CT uptake | Ongoing |

| | | | | | | | | | | | | | | |
|---|---|---|---|---|---|---|---|---|---|---|---|---|---|---|
| 4 | Retrospective multicenter study (US) | Morrisette et al, 2019 [36] | 1 | MRSA | LVAD | No | Yes | Daptomycin | 1500 mg, 2 doses post-discharge | Not specified | Not specified | No | Therapeutic failure due to uncontrolled infection. Readmission and further antibiotic treatment. | |
| 5 | Multicenter, observational retrospective study (Spain) | Hidalgo-Tenorio et al, 2019 [10] | 23 | MSSA 4, *S. epidermidis* 9, MRSA 3, *E. faecalis* 1, CNS 3, *S scleiferi* 1, *S bovis* 1, *S. lugdunensis* 1 | PVE 15, CIED 8 | Yes in 10 out of 15 PVE cases | No | Various antibiotic treatment | 1000 mg followed 500 mg weekly or 1500 mg followed 1000 mg everyr 2 weeks | No | 1 to 4 infusion (maximus at 45 days) | 1 patient had renal failure | Clinical success in 21 patients (95%). Death occurred in 2 patients after 2 and 6 months respectively, not related with IE | 12 months |
| 6 | Single case report (USA) | Howard-Anderson et al, 2019 [37] | 1 | MSSA | LVAD | No | Yes | Cephalexin, doxycycline, trimethoprim/sulfamethoxazole, linezolid | 1500 mg weekly, then 1500 mg every two week | No | 8 months | No | Clinical success. Driveline exit site infection to other organism; the patient required a switch to oral therapy. No further cultures identifying MSSA | Not specified |
| 7 | Single-center retrospective case series (US) | Deida et al, 2020 [38] | 2 | MRSA – 2 cases, polimicrobial gram positive - 1 case | PVE 2 CIED 1 | Not specified for the 2 PVE cases. Yes, for CIED | No | Various antibiotic treatment | 1500 mg single dose | No | Single dose | Not specified | Clinical success. Patients received a single dose of dalbavancin 7–10 days before the planned end date to facilitate earlier hospital discharge | 90 days |
| 8 | Single-center retrospective case series (Germany) | Hitzenbichler et al, 2020 [29] | 4 | MRSA 1, *E. faecalis* 1, *E. faecium* 2 | PVE 1 LVAD 2 TAVI 1 | No | Yes | Vancomycin, rifampicin, daptomicin, piperacillin | 1000 mg followed by 500 weekly or 1500 mg followed by 1000 mg biweekly (one patient | No | 1-12 months | Mild skin rash in 1 case | Clinical success, 1 patient is still undergoing treatment with one year of follow up and improvement | Ongoing |

| | | | | | | | | | | | | | | |
|---|---|---|---|---|---|---|---|---|---|---|---|---|---|---|
| | | | | | | | | | received 375 mg, adjusted for renal function) | | | | | |
| 9 | Single-centre retrospective study of IE (Italy) | Ciccullo et al, 2020 [28] | 1 | MRSA | PVGI | No | No | Rifampicin, vancomycin | 1000 mg following 1000 mg or 500 mg every week | No | 10 weeks - 10 doses | No | Clinical success. The treatment duration was extended based on PET–CT results that showed minimal residual uptake, for over 6 doses | 6 months |
| 10 | Single-centre retrospective study (Italy) | Durante-Mangoni et al, 2021 [24] | 7 | *Staphylococcus aureus* - 1 Coagulase-negative *Staphylococci* - 2 *Streptococcus* spp. - 3 *Enterococcus faecium* - 2 *Enterococcus faecalis* - 1 | PVE 4, TAVI 1, CIED 2 | 5 patients underwent surgery for IE, of whom 3 before and 2 after dalbavancin | 3 patients received prolonged dalbavancin to sterilize prosthetic material not removed due to high surgical risk | Various antibiotic treatment | 1500 mg or 1000 mg followed by 0–6 weekly doses | No | 1–6 weeks | Skin rash in 1 patient; mild liver enzyme increases in 2 patients, thrombocytopenia in 3 patients | Clinical success in 4 patients. 2 patients experienced relapse of IE after dalbavancin (CIED e PVE). No 90-day mortality was reported due to the initial infection | Median follow-up was 2 months |
| 11 | Single-center retrospective case series (UK) | Guleri et al, 2021 [21] | 4 | *Staphylococcus aureus* - 1 *Streptococcus* spp. -1 *Enterococcus faecalis* - 1 | PVE 1 TAVI 2, CIED 1 | 2 patients underwent surgery | No | Various antibiotic treatment | 1500 mg single dose or twice, 1 week apart | No | 1- 2 weeks, single or 2 doses | No | Clinical success | 12 months, |
| 12 | Multicenter retrospective study (Spain) | Ruiz-Sancho et al, 2023 [32] | 6 | MSSA 2, *S. gallolíticus* 1, *S intermedius* 1, *E. faecium* 1, unknown 1 | PVGI 5, overlap of infection sites1 | No | Yes | Various antibiotic treatment | 1000 mg followed 500 mg weekly or 1500 mg followed 1500 mg every 2 or 3 weeks | No | 14 - 118 weeks | Asthenia (1) moderate liver and kidney injury (1) | Clinical success in 5 patients (83.3%). Therapeutic failure due to uncontrolled infection in 1 patient PVGI. The patient died 3 months after dalbavancin was | Not specified |

| | | | | | | | | | | | | | | |
|---|---|---|---|---|---|---|---|---|---|---|---|---|---|---|
| | | | | | | | | | | | | | initiated. In 1 patient dalbavancin was discontinued due to liver injury | |
| 13 | Single center retrospective case series (US) | Rowe et al, 2023 [35] | 8 | MRSA 4, MSSA 1, polimicrobial Gram positive 3 | LVAD 8 | No | Yes | Various antibiotic treatment, in one patient dalbavancin was used as primary treatment | Biweekly or weekply switched to biweekly after 2 mo | No | 15 -39 weeks, still ongoing for 3 patients | No | Clinical success in 3/8 patients, 5/8 experienced a breakthrough Gram-positive infection despite suppressive dalbavancin therapy | Ongoing |
| 14 | Single center retrospective case series (Italy) | Gallerani et al, 2023 [12] | 14 | MSSA 4, MRSA 1, *E. faecalis* 1, *S. capitis*, *Gemella morbillorum* 1, *S. mitis*/*oralis*, *S.* lugdunensis 1, *S. sanguinis* 1, *S. pasteurianus* 1, *C. striatus* 1, unknown 1 | CIED 3, PVE 8, PVGI 7, overlap of infection sites 4 | 6 patients underwent surgery, only 4 achieved complete sources control | Partial | Various antibiotic treatment | 1500 mg plus 1500 mg, then according to TDM, or 1500 mg plus 1500 mg, or 1500 mg plus 1500 mg then 1000 mg every 14 days | Yes, 9 out of 14 | 1 to 45 weeks (1 patient still in ongoing therapy) Among patients managed with TDM-guided strategy, dalbavancin infusion intervals ranged from 4 to 9 weeks | Rash in 1case. Impaired renal function in 1 case | Clinical success rate of 77%. All failures occurred in patients with retained implant | 65 weeks (interquartile range: 23–144 weeks) |
| 15 | Single center retrospective case series (US) | Mansoor et al, 2023 [26] | 10 | *C. striatum* 6, MRSE 1, polymicrobial Gram positive 1 | LVAD 10 | | Yes | Various antibiotic treatment | 1000–1500 mg every 2 weeks, 375–500 mg weekly | No | 13 to 124 weeks (max 2 years and 4 months) still ongoing for 6 patients | No | Clinical success in 6 patients. In 2/10 breakthrough Gram-positive infection despite suppressive dalbavancin therapy. 1 underwent a heart transplant after 35 weeks, 1 died from | Ongoing |

| | | | | | | | | | | | | | | |
|---|---|---|---|---|---|---|---|---|---|---|---|---|---|---|
| | | | | | | | | | | | | | out-of-hospital cardiac arrest after 39 weeks | |
| 16 | Single center retrospective case series (Italy) | Pallotto et al, 2024 [34] | 4 | MSSA1, MRSA 1, MRSE 2 | LVAD 1, PVGI 1, PVE 2 | | Yes | Various antibiotic treatment | l500 mg at day 1 and 8 and then every 4weeks | No | 12 to 114 weeks | No | Clinical success in 3 patients. 1 patient with endocarditis experienced a relapse, but the infection was controlled with dalbavancin | Ongoing |
| 17 | Single center retrospective case series (Spain) | Salinas-Botrán et al, 2024 [23] | 11 | MRSA1, MSSE 3, CoNS 4, *E faecalis* 1, *S. viridans* 1, *S. agalactiae* 1 | PVE 8; CIED 2; PVGI | 8 patients underwent surgery | partial, 3/11 | Various antibiotic treatment | Doses varied between 500 mg, 1000 mg, and 1500 mg, administered either as a single dose or weekly or biweekly | No | 1 to 12 weeks | No | Clinical success in 9/11 patients (81.8%). 2 PVE patients worsened during the dalbavancin treatment and required valve replacement. 1 vascular graft infection patient on compassionate dalbavancin therapy | 17 weeks (1-64 weeks median) |
| 18 | Single case report (Spain) | Cepeda et al, 2024 [33] | 1 | *S. epidermidis* | TAVI | No | Yes | Daptomycin | 1500 mg every 2 weeks | No | 5 months | No | Therapeutic failure with emerging resistance dalbavancin, therapy switched to tedizolid | |

PVE: prosthetic valve endocarditis; CIED: cardiac implantable electronic device infections; LVAD: left ventricular assist device infections; PVGI: Prosthetic vascular graft infections; IE: infective endocarditis; DBV: dalbavancin; MSSA: meticillin-sensitive *Staphylococcus aureus*; MRSA: meticillin-resistant *Staphylococcus aureus*; CoNS: Coagulase-Negative Staphylococci; TDM: Therapeutic drug monitoring, PET–CT: Positron Emission Tomography/Computed tomography.

**Table S3.** Studies included in the review on patients treated with oritavancin for cardiovascular prosthetic infections.

| | Study type | Reference | No. of patients | Organism(s) | Type of Infection | Prosthetic removal | Indication for suppressive antibiotic therapy | Antibiotics used prior to dalbavancin | Oritavancin dose | Therapeutic drug monitoring | Oritavancin duration | Adverse events/ failure of dalbavancin | Failure of Oritavancin treatment | Follow-Up (Weeks) |
|---|---|---|---|---|---|---|---|---|---|---|---|---|---|---|
| 1 | Single case report Suppressive therapy (US) | Johnson et al, 2015 [27] | 1 | *E. faecium* VRE | PVE | Yes | No | Various antibiotics | 1200 mg every other day for 3 doses, then weekly for 6 weeks; after the relapse, surgery and then oritavancin 1200 mg biweekly for 10 weeks | Yes | 17 weeks | No | Relapse after 8 days with persistent bacteremia, leading to valve surgery due to prosthetic valve infection and mitral endocarditis. Clinical success after valve replacement. | 22 months |
| 2 | Single center retrospective case series of patients receiving oritavancin (US) | Shulz et al, 2017 [30] | 1 | *Staphylococcus lugdunensis* | PVGI | No | Yes | Cefazolin | 1200 mg, then 800 mg weekly for 11 weeks, then 1200 mg following an 11-day interval, then 800 mg for 5 weeks | No | 17 weeks | No | Clinical success. Oritavancin chosen as palliative, suppressive treatment following successful treatment with cefazolin | Not specified |

PVE: prosthetic valve endocarditis; PVGI: Prosthetic vascular graft infections; VRE: Vancomycin-resistant *Enterococcus*. PVE: prosthetic valve endocarditis; TDM: Therapeutic drug monitoring.

**Table S4.** Risk of bias appraisal of the included studies using the Joanna Briggs Institute (JBI) Critical Appraisal Checklist

| Case Series | | | | | | | | | | |
|---|---|---|---|---|---|---|---|---|---|---|
| | **Were there clear criteria for inclusion in the case series?** | **Was the condition measured in a standard, reliable way for all participants included in the case series?** | **Were valid methods used for identification of the condition for all participants included in the case series?** | **Did the case series have consecutive inclusion of participants?** | **Did the case series have complete inclusion of participants?** | **Was there clear reporting of the demographics of the participants in the study?** | **Was there clear reporting of clinical information of the participants?** | **Were the outcomes or follow up results of cases clearly reported?** | **Was there clear reporting of the presenting site(s)/clinic(s) demographic information?** | **Was statistical analysis appropriate?** |
| Morrisette [36] | Yes | Yes | Yes | Yes | Yes | Yes | Yes | Yes | Yes | Yes |
| Tobudic [22] | Yes | Yes | Yes | No | Yes | Yes | Yes | Yes | Yes | Not applicable |
| Salinas-Botrána [23] | Yes | Yes | Yes | No | Yes | Yes | Yes | Yes | Yes | Yes |
| Guleri [21] | Yes | Yes | Yes | No | Yes | Yes | Yes | Yes | Yes | Yes |
| Pallotto [34] | Yes | Yes | Yes | No | No | Yes | Yes | Yes | Yes | Yes |
| Mansoor [26] | Yes | Yes | Yes | No | Yes | Yes | Yes | Yes | Yes | Yes |
| Gallerani [12] | Yes | Yes | Yes | Yes | Yes | Yes | Yes | Yes | Yes | Yes |
| Rowe [35] | Yes | Yes | Yes | Yes | Yes | Yes | Yes | Yes | Yes | Yes |
| Ruiz-Sancho [32] | Yes | Yes | Yes | Yes | Yes | Yes | Yes | Yes | Yes | Yes |
| Durante-Mangoni [24] | Yes | Yes | Yes | No | Yes | Yes | Yes | Yes | Yes | Not applicable |
| Hitzenbichler [29] | Yes | Yes | Yes | No | Yes | Yes | Yes | Yes | Yes | Not applicable |
| Deida [38] | Yes | Yes | Yes | No | Yes | No | Yes | Yes | Yes | Yes |
| Hidalgo-Tenorio [10] | Yes | Yes | Yes | Yes | Yes | Yes | Yes | Yes | Yes | Yes |
| Schulz [30] | Yes | Yes | Yes | Yes | Yes | Yes | Yes | Yes | Yes | No |

| Case Reports | | | | | | | | |
|---|---|---|---|---|---|---|---|---|
| | **Were patient's demograpshic characteri** | **Was the patient' history clearly describe** | **Was the current clinical condition of the** | **Were diagnostic tests or assessment** | **Was the intervention(s) or treatment procedure** | **Was the post-intervention clinical** | **Were adverse events (harms) or** | **Does the case report provid** |

| | stics clearly described? | d and presented as a timeline? | patient on presentation clearly described? | methods and the results clearly described? | (s) clearly described? | condition clearly described? | unanticipated events identified and described? | e takeaway lessons? |
|---|---|---|---|---|---|---|---|---|
| Howard-Anderson [37] | Yes | Yes | Yes | Yes | Yes | Yes | Yes | Yes |
| Kussmann [25] | Yes | Yes | Yes | Yes | Yes | No | No | Yes |
| Johnson [27] | Yes | Yes | Yes | Yes | Yes | Yes | Yes | Yes |
| Cepeda [33] | Yes | Yes | Yes | Yes | Yes | Yes | Yes | Yes |
| Cicculo [28] | Yes | Yes | Yes | Yes | Yes | Yes | Yes | Yes |
| Spaziante [31] | Yes | Yes | Yes | Yes | Yes | Yes | Yes | Yes |
